# Allele discovery of ten candidate drought-response genes in Austrian oak using a systematically informatics approach based on 454 amplicon sequencing

**DOI:** 10.1186/1756-0500-5-175

**Published:** 2012-04-03

**Authors:** Andreas Homolka, Thomas Eder, Dieter Kopecky, Maria Berenyi, Kornel Burg, Silvia Fluch

**Affiliations:** 1 Health and Environment Department, AIT Austrian Institute of Technology, Tulln, A-3430, Austria

## Abstract

**Background:**

Rise of temperatures and shortening of available water as result of predicted climate change will impose significant pressure on long-lived forest tree species. Discovering allelic variation present in drought related genes of two Austrian oak species can be the key to understand mechanisms of natural selection and provide forestry with key tools to cope with future challenges.

**Results:**

In the present study we have used Roche 454 sequencing and developed a bioinformatic pipeline to process multiplexed tagged amplicons in order to identify single nucleotide polymorphisms and allelic sequences of ten candidate genes related to drought/osmotic stress from sessile oak (*Quercus robur*) and sessile oak (*Q. petraea*) individuals. Out of these, eight genes of 336 oak individuals growing in Austria have been detected with a total number of 158 polymorphic sites. Allele numbers ranged from ten to 52 with observed heterozygosity ranging from 0.115 to 0.640. All loci deviated from Hardy-Weinberg equilibrium and linkage disequilibrium was found among six combinations of loci.

**Conclusions:**

We have characterized 183 alleles of drought related genes from oak species and detected first evidences of natural selection. Beside the potential for marker development, we have created an expandable bioinformatic pipeline for the analysis of next generation sequencing data.

## Background

White oaks are native to Europe, Asia, North Africa and North America, and include sessile oak, pedunculate oak, pubescent oak (*Q. pubescens*) and bur oak (*Q. macrocarpa*) among their most prominent species [1]. About two percent of the Austrian forests harbour oak trees which correspond to an area of 66,000 hectare. *Q. petraea* and *Q. robur* are the predominant species while *Q. cerris* (Turkey oak) and *Q. pubescens* play only a minor role [2]. In Austria as well as in Europe oak species colonize huge areas with vastly differing climatic conditions.

Rise of temperatures and shortening of available water as result of predicted climate change will impose significant pressures on long-lived forest tree species like European white oak. According to the latest predictions, we expect the average global surface temperature to increase by a maximum of 6.4°C within the next 90 years [3] leading to a higher frequency of severe drought events. But it is well known that different species diverge in their ability to resist drought induced damages and even within a species there is tremendous variability [4,5]. Inter- as well as intraspecific allelic diversity is the key element of a plants potential to adapt to a changing environment and tolerance towards drought stress. There is an increased demand for molecular tools helping to describe these variations and to prepare forestry for future challenges.

Molecular markers are the first choice for plant research and breeding [6]. Using them as landmarks, genetic maps can be established and subsequently used for identification of traits controlled by different genes (quantitative trait loci). Marker assisted selection provides breeders with an efficient tool for identifying desired phenotypes in large populations. Beside their use in breeding, molecular markers are highly valuable for population genetics permitting evolutionary studies and population structure interference.

Single nucleotide polymorphisms (SNPs) are commonly used for functional diversity assessment. Although they are highly abundant in the human genome and every SNP locus could potentially serve as utile marker, there are still few studies dealing with a high number of SNPs in plants [7]. Latest advances in human and animal genome analysis have created several sophisticated technologies which are capable to analyze millions of SNPs in reasonable time and with low costs [6] and can be easily transferred to applications in plants. SNP discovery technologies include bioinformatic mining of expressed sequence tag (EST) databases [8], array based methods [9], comparison of whole genomes [10] and the application of next generation sequencing (NGS) for amplicon resequencing [11]. Recently developed sequencing technologies summarized as next generation sequencing have already replaced traditional methods for detecting polymorphisms in genomes [12].

Roche 454 sequencing technology [13] is based on single strand amplification with emulsion polymerase chain reaction followed by pyrosequencing. Average readlengths of around 400 bp and high achievable coverage makes this technology well suited for discovery of SNPs and even detection of rare alleles. Short oligonucleotide barcodes can be used to tag individual sequences and enable the parallel analysis of several targets which has been successfully demonstrated in different species [14-16]. Multiplexing capabilities enable large studies including several hundred individuals with a high coverage for each sample. Therefore extensive cloning procedures to identify both haplotypes of diploid individuals as used in Sanger sequencing or generation of inbred lines [17] can be avoided. Bioinformatic haplotype interference with parsimony [18] or maximum-likelihood methods [19] is no longer necessary because each haplotype will be covered by a sufficient number of reads. The present paper describes the discovery and characterization of alleles in ten drought stress related genes originating from two oak species growing in Austria based on multiplex 454 amplicon sequencing and the development of a bioinformatic analysis pipeline.

## Results

### Processing of 454 sequencing data

**Figure 1:**
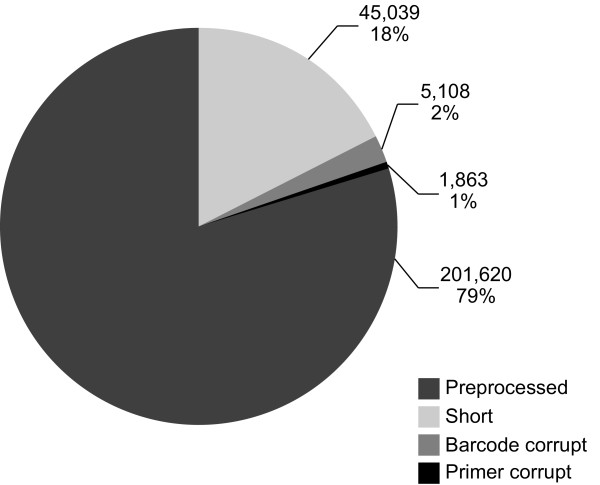
**Distribution of all reads after preprocessing.** Amount of reads lost due to technical reasons including sequences shorter than 90 bp, reads with corrupt barcode or primer and remaining reads are displayed.

Raw data with a total number of 253,630 reads comprising 57.8 Mbp with an average length of 227.85 bp was delivered by the sequencing company (Table 1). Average 454 quality score of the provided sequences was 22.95 (median 25.19) with a maximum of 37.93 and a minimum of 5.50. We removed 45,039 reads shorter than 90 bp (Figure 1) with an average length of 67.93 bp. A total number of 298 sequences longer than 420 bp with an average length of 447.21 bp and a maximum of 705 bp were trimmed. Average 454 quality score after removal of long and short sequences increased to 27.78 (median 28.24). Barcode sequences were not readable in 5,108 reads which were therefore discarded. Additionally we identified 1,863 reads with corrupt internal primers which were excluded from further analysis (Figure 1). After preprocessing 201,620 reads consisting of 53 Mbp sequence information with an average length of 230.35 bp remained for allele detection (Table 1).

**Table 1:** Comparison between raw data and preprocessed reads

**Pool**						
		**Received**			**Preprocessed**	
	**Number of reads**	**Base pairs (Mbp)**	**Average length**	**Number of reads**	**Base pairs (Mbp)**	**Average length**
Oak 1	30,207	5.72	189.45	22,826	5.11	200.96
Oak 2	38,797	8.55	220.43	29,290	7.71	239.25
Oak 3	31,731	6.70	211.29	24,336	6.08	229.92
Oak 4	31,874	7.63	239.31	26,063	7.10	248.58
Oak 5	33,645	8.68	258.07	30,619	8.38	180.76
Oak 6	30,008	6.95	231.72	23,650	6.39	246.25
Oak 7	29,307	7.07	241.34	23,909	6.61	252.14
Oak 8	28,061	6.49	231.16	20,927	5.61	244.92
Total	253,630	57.80	227.85	201,620	52.98	230.35

During homopolymer (HP) correction in SCARF [20] 17,287 additional reads were removed because the software was not able to align them to the Sanger reference [21]. On average 18 individuals per locus were removed from the analysis (Table 2) because of technical errors including incorrect amplification, short reads, read quality and corrupted barcodes or internal primers. A maximum number of 28 lost individuals was found in *BMY7* while in *PIP1E* we only lost 7 individuals.

**Table 2:** Recovery statistics and individual coverage

**Gene**						
	**Technical**	**Coverage**	**Remaining**			**Average**
	**loss**	**loss**	**Individuals**		**Reads**	**coverage**
*ARF16*	27	18	291	(86.6%)	9,327	32.05
*BMY7*	28	16	292	(86.9%)	13,189	45.17
*DHN2*	22	99	n.a.		n.a.	n.a.
*ERD8*	8	9	319	(94.9%)	11,941	37.43
*LEA14*	25	2	309	(92.0%)	16,102	52.11
*LTP*	18	5	313	(93.2%)	20,409	65.20
*PER64*	11	2	323	(96.1%)	22,628	70.06
*PIP1E*	7	4	325	(96.7%)	11,364	34.97
*RD26*	15	9	312	(92.9%)	9,898	31.72
Average	18	18	310	(92.3%)	14,357	46.09
Total	161	164	2,484		114,858	

### Polymorphic sites and allele identification

**Figure 2:**
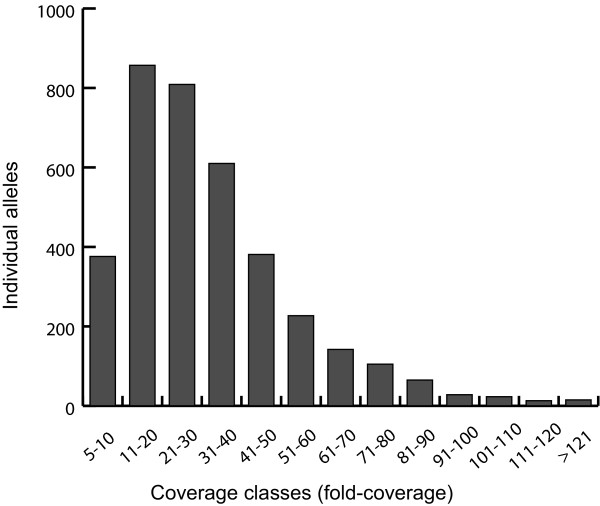
**Allelic coverage distribution.** Number of alleles found in different classes of fold coverage is displayed.

Within the sequences of *GLP3A* we detected more than 2 alleles per individual. A minimum number of one allele and a maximum number of 12 alleles per individual with an average of 2.6 were found. *GLP3A* was excluded from further analysis because we suspected the amplification of a gene family or pseudogenes. Including individuals with less than four reads covering 70 % of the Sanger reference 65,181 reads were removed from the 201,620 reads remaining after preprocessing. Due to low coverage an average number of 18 individuals had to be excluded from further analysis with a maximum number of 99 in *DHN2* and a minimum number of two found in *LEA14* and *PER64* (Table 2). *DHN2* was not included in the further analysis because we considered the number of individuals lost due to low coverage (99) as too high. Excluding *DHN2*, the average percentage of recovered individuals was 92.3%. On average each gene fragment was covered by 46.09 reads. In total we detected 158 polymorphic sites [SNPs and Insertions/Deletions (InDels)] among the eight remaining candidate genes (Table 3) with an average of 19.75 sites per gene. The highest number was found in *LEA14* and *LTP* (34), whereas a minimum number of seven polymorphic sites was detected in *PER64*. On average 6.7 polymorphic sites occur each 100 bp with a minimum of 2.8/100 bp in *ARF16* and a maximum of 13.7/100 bp in *LTP*. We found an average number of 22 alleles per gene with a maximum of 52 in *BMY7* and a minimum of ten in *ARF16*, *PER64* and *RD26*. The total number of detected alleles was 183. Sequences of alleles as well as genotypes of the analyzed individuals can be found in Additional file 1. Each allele of an individual was covered by an average of 32.64 reads (median 28, Figure 2). Total number of effective alleles was 23.561 with a minimum of 1.253 (*RD26*), a maximum of 6.656 (*BMY7*) and a mean value of 2.945 (Table 3). Values for expected heterozygosity ranged from 0.202 (*RD26*) to 0.851 (*BMY7*) with an average number of 0.545. Regarding observed heterozygosity, a minimum of 0.115 in *RD26* and a maximum of 0.640 in *LEA14* was detected. The mean value of observed heterozygosity was found to be 0.347. In all genes expected heterozygosity exceeded the observed. All loci showed significant departure from Hardy-Weinberg expectations (HWE, Guo and Thompson’s exact test [P < 0.05]). Null alleles were present in a high frequency (above 5%) in all loci observed. Tests for linkage disequilibrium (LD) revealed significant disequilibrium (P < 0.01) within six pairwise combinations of loci ( Additional file 2: Table S2).

**Table 3:** Polymorphisms detected

**Gene**	**S**	**N**_**a**_	**N**_**e**_	**H**_**o**_	**H**_**e**_	**F**_**n**_	**HWE**	**Mutations/100 bp**
*ARF16*	10	10	1.667	0.249	0.404	0.134	0.000	2.75
*BMY7*	19	52	6.656	0.481	0.851	0.189	0.030	5.97
*ERD8*	26	27	2.602	0.551	0.619	0.112	0.000	7.24
*LEA14*	34	49	5.079	0.640	0.803	0.095	0.000	12.23
*LTP*	34	13	2.849	0.260	0.648	0.228	0.000	13.71
*PER64*	7	10	1.817	0.209	0.444	0.182	0.000	3.37
*PIP1E*	15	12	1.638	0.274	0.391	0.111	0.000	4.79
*RD26*	13	10	1.253	0.115	0.202	0.115	0.000	3.61
Average	19.75	22.88	2.945	0.347	0.545	0.146	0.004	6.71
Total	158	183	23.561					

S Number of polymorphic sites, N_a_ number of alleles, N_e_ effective number of alleles, H_o_ observed heterozygosity, H_e_ expected heterozygosity, F_n_ frequency of null alleles, HWE test for deviation from Hardy-Weinberg equilibrium (p-value)

## Discussion

Several studies describe the *in silico* detection of SNPs from the growing amount of EST data available [22,23]. However this approach is likely to be biased by the small number of individuals coming from a limited amount of populations [24]. To overcome this limitation we made use of high throughput sequencing techniques and a bioinformatic pipeline. 454 Sequencing technology developed by Roche has been chosen to analyze coding regions of drought responsive candidate genes. The main of advantage of this technique is the high number of reads generated leading to adequate coverage of each allele. Major drawbacks include the preferential amplification of short sequences and the high abundance of reading errors after homopolymer stretches. To cope with these problems we have developed a highly customizable pipeline which integrates preprocessing, statistics and error correction for 454 amplicon sequencing data and can be used to resolve multiplexed samples and to detect variants in a large number of sequences.

Different studies based on molecular markers have been conducted in oak. But the majority was investigating the population history of *Quercus* spp. [25] and hybridisation between *Q. robur* and *Q. petraea*[26]. Only Derory *et al.*[27] and Quang *et al.*[28] used SNPs to assess allelic diversity of candidate genes. So far, this is the first study which aimed at detecting alleles of drought stress related genes of oak. With the aid of the bioinformatic pipeline presented above were able to detect a total amount of 183 alleles within eight genes of 336 oak individuals growing in Austria. The amount of alleles is comparable to results from white spruce where 173 alleles were detected in six loci from 283 individuals growing in Alaska [29]. The average number of 6.71 mutations per 100 bp is higher or at least in the same range than in other plant species. Frequencies of 2.96 to 3.70, 3.85 or 3.83 to 7.30 have been discovered in maize [30], black poplar [31] and eucalyptus [16] which shows that the number of mutations strongly varies between species and gene analyzed. A high level of heterozygosity is present within *ERD8* and *LEA14* which points towards a large amount of genetic variability. Regarding the large number of individuals sampled across the heterogenic climatic area of Austria, a high variability is expected especially in genes like *ERD8* and *LEA14* which are directly involved in processes regulating drought response.

The high number of discovered alleles will be used as valuable source for association studies between allele frequencies and environmental variables in connection with drought stress. SNPs discovered in this study can be used to describe the genetic diversity present in two Austrian oak species and to develop molecular markers for drought tolerance if natural selection can be proven for some loci. Significant deviation from Hardy-Weinberg equilibrium as well as lower values of observed heterozygosity than expected at all loci may be the first evidence for natural selection. The deviations could partly be a result of sampling, be explained by the presence of null alleles (Table 3) or might arise from selective pressure on the coding regions. Although varying among genes, low levels of observed heterozygosity and an excess of homozygotes at *RD26* support the latter hypothesis as well as the presence of linkage disequilibrium between some pairs of loci. This might either occur due to epistatic natural selection where favourable combinations of genes are linked and function together as supergenes [32] or several other factors including gene flow which is well documented among several oak species [27,33,34]. Present linkage between pairs of loci is the basis of association studies which can help to identify alleles occurring more frequently in plants exposed to dry conditions. Linkage between several loci of drought reactive genes is generally expected as drought response is under control of a huge regulatory network [35,36]. However, this approach is limited by the fact that rapid decay of LD is commonly observed in forest tree species [37,38] increasing the marker density needed for successful association studies.

Considering these findings, it points out clearly that the available dataset should be subject of further analysis like decay of LD, estimation of genetic diversity or differentiation of populations. To evaluate if selective forces were acting, different neutrality test as well as detection of outlier loci will be necessary. To evaluate if these deviations were only generated by demographic processes the results should be related to neutral markers like microsatellites found in chloroplasts (cpSSRs). If the presence of loci under selection and their association with climatic variables like temperature and precipitation could be proven, development of molecular markers will be possible. Given the predicted climate change and the resulting pressure on sessile organisms, functional markers might be a valuable tool for forestry and a basis for marker assisted selection of drought tolerant genotypes. Additional benefit arises from the fact that two different oak species were included in the analysis. Although they are mainly sympatric, *Q. petraea* is not as susceptible to drought induced damages as *Q. robur*. If species specific alleles can be discovered and related to environmental conditions the genetic basis of this benefit may be revealed.

## Conclusions

Given next generation sequencing data of ten drought stress related genes originating from different oak species growing in Austria, we were able to discover new SNPs and characterize 183 alleles. In order to obtain these results, we have developed a semiautomatic analysis pipeline based on freely available tools and scripts. This pipeline can be fully automated and provided with a graphical user interface to make it more valuable for the scientific community. First analysis of the genetic data provides evidence of natural selection acting on the genes which makes them a target for future evolutionary studies and a potential source for molecular marker development. The alleles discovered will definitely help to understand drought adaptation processes acting in forest tree species.

### Availability of supporting data

The raw data supporting the results of this article are available in the Dryad repository,http://dx.doi.org/10.5061/dryad.83gf113b. The data sets supporting the results of this article are included within the article and its additional files.

## Methods

### Plant material and DNA isolation

Genomic DNA from 336 oak individuals collected across Austria [39,40] was extracted with the DNeasy Plant Mini Kit (Qiagen) according to the manufacturer’s instructions. Microarray experiments with long-term drought stress applied to oak clones [21] provided the basis for selection of ten putative candidate genes for drought adaption (Table 4).

**Table 4:** Summary data of candidate genes

**Gene**	**Annotation**	**Accession**	**Primer pairs**	**T**_**a**_**(°C)**	**Length**
*ARF16*	Auxin responsive factor 16	FP033974	Fw: 5′-M13Fw-CAGTTGATGTTGGTTGGACA-3′	55	363
			Re: 5′-M13Re-ACAATAAACAAATGCTACTCA-3′		
*BMY7*	Beta-amylase 1	FP061723	Fw: 5′-M13Fw-TGCATTGCCTCGTTATGATG-3′	59	318
			Re: 5′-M13Re-TGAAACCATGGGTGAAACCT-3′		
*DHN2*	Dehydrin 2	FP045825	Fw: 5′-M13Fw-AGCAGCAGCAAGGTC-3′	62	309
			Re: 5′-M13Re-CCTTGATCTTCTCCATCACTCC-3′		
*ERD8*	Heat shock cognate protein 80	FP041070	Fw: 5′-M13Fw-AGAATGACAAATCAGTGAAGGA-3′	59	359
			Re: 5′-M13Re-CGCATCAAAGCAATAGCAAA-3′		
*GLP3A*	Germin-like protein 3A	FP024682	Fw: 5′-M13Fw-GGAGACCTGGGCTTGAACTA-3′	59	344
			Re: 5′-M13Re-TGTCAATGGCCTTGGAATTT-3′		
*LEA14*	Desiccation protectant protein Lea14	FP049285	Fw: 5′-M13Fw-TGTGCAGATGGGGACATAGA-3′	57	278
			Re: 5′-M13Re-TGCAACAAAAATTGAAGATAGGAA-3′		
*LTP*	Prolin-rich protein	FP031479	Fw: 5′-M13Fw-GCTTCTTAGTGCTTGCCAAAA-3′	60	248
			Re: 5′-M13Re-TCAGATTTCAGCCCATCTGAG-3′		
*PER64*	Peroxidase 64	FP051623	Fw: 5′-M13Fw-CAACCACCACAGCATTTGAC-3′	62	208
			Re: 5′-M13Re-CCTAATCTCTTGCCCACCAG-3′		
*PIP1E*	Aquaporin PIP1-2	FP040796	Fw: 5′-M13Fw-CTGTGGTAAAGGGCTTCCAA-3′	58	313
			Re: 5′-M13Re-ACACTGCAAACCCAATAGGC-3′		
*RD26*	Responsive to desiccation 26	FN712698	Fw: 5′-M13Fw-AATTATTGGTGACATTGATTTG-3′		
			Re: 5′-M13Re-GGGGCTTTTCCAATATAGAAT-3′	54	360

Accession GeneBank accession number, T_a_ annealing temperature of gene specific primers

### Amplification of candidate genes

Sequencing adaptors and barcodes were attached to the gene of interest following a modified two-step approach used by Schuelke [41]. In the first step, genespecific primer pairs (internal primers) with M13-tails were used to amplify regions of interest. Internal primers targeting regions with a minimum of 200 and a maximum of 375 bp were planned with Primer3 [42] using default settings. M13Fw (5′-TGTAAAACGACGGCCAGT-3′) and M13Re (5′-CAGGAAACAGCTATGACC-3′) were synthesized to the 5′-end of the internal primers. Amplicons for 454 sequencing were generated from 20 ng of template DNA. PCR reactions were performed in 25 μl total volume using 5 μl 5 x HOT FIREPol® Blend Master Mix 12.5 mM MgCl_2_ without dye (Solis BioDyne) and 3 pmol of each primer. A three-step PCR program consisting of 15 min. initial denaturation at 95°C followed by 32 cycles denaturation at 95°C for 30 sec., annealing at a temperature of 68°C for 45 sec., extension at 72°C for one minute and a final extension at 72°C for 8 minutes was used.

Forward primer for the second amplification consisted of the 454 sequencing adaptor A (5′-CCATCTCATCCCTGCGTGTCTCCGACTCAG-3′) linked to a hexanucleotide barcode sequence corresponding to the amplified individual and the M13Fw sequence. Reverse primers consisted of the 454 sequencing adaptor B (5′- CCTATCCCCTGTGTGCCTTGGCAGTCTCAG-3′) and the M13Re sequence. HOT FIREPol® Blend Master Mix and a primer concentration according to Table 5 was used for the amplification including an initial denaturation step of 95°C for 15 minutes. Subsequent cycling comprised denaturation at 95°C for 20 sec., annealing at 55°C for time given in Table 5 and extension at 72°C for 15 seconds. The number of cycles was depending on the gene region amplified and can be found in Table 5. Product size and concentration was verified with gel electrophoresis on 1.0% agarose gels.

**Table 5:** PCR settings

**Gene**	**Annealing time (sec.)**	**Cycles**	**Primer concentration (pmol)**
*ARF16*	20	25	4
*BMY7*	25	26	4
*DHN2*	25	27	3
*ERD8*	20	28	4
*GLP3A*	25	27	3
*LEA14*	25	32	4
*LTP*	25	26	3
*PER64*	25	28	4
*PIP1E*	20	26	4
*RD26*	25	26	3

### Multiplexed 454 amplicon sequencing

PCR products of forty-two individuals per gene were pooled resulting in a total number of 80 pools. Cleaning was performed after pooling with the QIAquick PCR purification kit (QIAGEN) according to the manufacturer’s instructions. Concentrations of these pools were measured on Nanodrop (Thermo Scientific) and adjusted to 30 ng/μl adding TE buffer pH 8.0. PCR products of the same individuals originating from different genes were combined and sent for sequencing which was carried out by GATC Biotech in an 8 gasket format run on the Genome Sequencer FLX system (454 Life Sciences) with Titanium chemistry.

### Bioinformatic analysis pipeline

**Figure 3:**
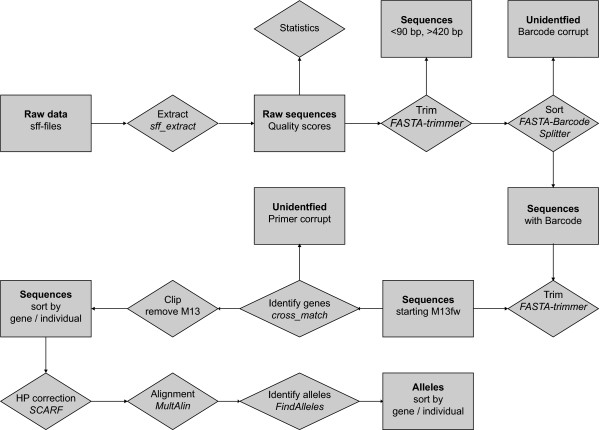
Flow chart of the bioinformatic pipeline developed for the analysis of a 454 amplicon resequencing experiment using freely available tools and custom Perl scripts.

A semiautomatic pipeline for allele identification was developed using several publicly available software tools. The general workflow is shown in Figure 3. Sequencing data delivered in sff format was extracted using *sff_extract* (http://bioinf.comav.upv.es/sff_extract/) with the clipping option set (*sff_extract -u -c input.sff -o output_raw*). Length and quality statistics of the sequencing runs were calculated in R 2.12.1 [43], which was also used for graphical representation. All sequences shorter than 90 base pairs were removed from the analysis with custom Perl or shell scripts because these only contained 16 bp with possible SNP information (90 bp less 30 bp adaptor, 6 bp barcode, 18 bp M13 and 20 bp genespecific primer). Sequences longer than 420 bp were trimmed using *FASTA-Trimmer* (*fastx_trimmer -l 420 -i input.long -o output_lenclipped.fasta*) which is part of the *FASTX-toolkit* (http://hannonlab.cshl.edu/fastx_toolkit/index.html) because a drop of quality below a threshold of 20 (454 quality score) was observed after this length. With the aid of the *FASTA-Barcode Splitter* which is also part of the FASTX-toolkit names of *Quercus* individuals were assigned to the corresponding reads with the exact match option set (*cat input_lenclipped.fasta | fastx_barcode_splitter.pl -bcfile Tags_Oak.txt -bol -exact -prefix BC_Oak_output/ -suffix “.fasta”*). For this, a textfile (Tags_Oak.txt) including the names of the individuals and the barcode sequences was used. Only perfect matching barcodes were considered for further analysis. After barcode identification, *FASTA-Trimmer* was used to remove the first six bases of each read (*fastx_trimmer -f 7 -i input.fasta -o output.trimmed*). To identify the gene primer *cross_match* (http://www.phrap.org/consed/consed.html) was applied with the minimum length of matched word set to eight and the minimum alignment score set to ten (*cross_match input.trimmed ../Internal_primer.fasta -minmatch 8 -minscore 10 > output.crossmatch*). Starting position of the cross_match hit was extracted from the output (*egrep “^ [0–9]” input.crossmatch | awk ‘$6 < 40 { printf(“%s\t%s\t-1\t%s\n”, $5, $6, $9) }‘ > output.crossmatch.filter*) and used in a custom Perl script to clip M13 primers which precede the gene primer sequences.

To cope with errors resulting from the misinterpretation of homopolymer stretches by 454 sequencing we made use of the assembly tool *SCARF*[20]. Sanger sequences [21] of the amplicons were used as references and homopolymer correction was turned on for a minimum length of 2 bp. Therefore homopolymer errors longer than the reference sequence will be trimmed to reduce read errors. Minimum percent identity and minimum hit score were set to 80 and 100, respectively (*./scarf -f input.fasta –r referrence.fasta -c T -l 2 -p 80 -s 100*). Alignment of the assembled reads was done with *MultAlin*[44] using the AltDNA symbol comparison table and the gap penalty at extremities parameter set to end (*ma -c: altdna.tab –x:1 input.clusters*). Reads with gaps larger than 10 bp were masked in the alignment using a bash script for detection and not considered for further analysis.

### SNP and allele detection

We created an extensive script (*FindAlleles*) using *BioPerl*[45] which is able to identify mutations and detect alleles. Msf alignment files produced by *MultAlin* serve as input and are read using the *Bio::AlignIO module*. In a next step three different consensus sequences are calculated using the *Bio::SimpleAlign module*: *consensus_iupac()*—consensus sequence using IUPAC ambiguity codes for DNA, *consensus_string()*—standard consensus sequence displaying bases which occur in plurality and *consensus_string(40)*—produces a standard consensus and marks positions with a lower percent-identity than 40 % with “?”. A column representation of all sequence reads in the alignment is created. Using the three consensus sequences created, each column is screened for possible allelic distribution or arbitrary nucleotide insertions or deletions created by 454 sequencing technology and both types are annotated with a tag. In a next step the script checks each column for an allelic distribution of a 40/60 ratio. This correction threshold value was identified by manually examining randomly chosen alignments. In the examined alignments reading errors were not present in more than ten percent of the reads. The 40/60 distribution might be created by two different nucleotides (nucleotide variation) or by the insertion or deletion of a nucleotide (InDel). If this ratio is found the position is considered as valid “allelic” distribution. If it is not found, it is considered as reading error and is automatically corrected with the nucleotide found in the Sanger reference if a previously set correction threshold of ten percent is reached. As *SCARF* only treats homopolymer errors longer than the length present in the reference, we implemented an additional routine to fill up homopolymers shorter than the reference. As we observed errors occurring already in homopolymers consisting of two nucleotides, the min homopolymer parameter which sets the amount of nucleotides in a row to trigger correction was set to one. Nucleotide positions exceeding the reference are ignored.

After the correction phase the allele identification procedure is started running again over each column. If a tag for allelic distribution is found, the process splits up the available reads into two subgroups and a recursive subroutine is started for each split alignment. Each subroutine receives the associated reads and continues with them. Reads shorter than 70 % of the reference were discarded. Then the tags are checked and split up again if an allelic distribution is found. After all subroutines are finished alleles are exported to a multiple fasta file as well as statistics about the number of reads accounting for their creation and a text file with the excluded reads are generated. A cluster for allele generation was considered as valid if at least five reads covered more than 70 % of the reference sequence derived from Sanger sequencing. Number of alleles, observed and expected heterozygosities, deviation from Hardy-Weinberg equilibrium and linkage disequilibrium were calculated with Genepop 4.0 [46]. Frequency of null alleles was estimated with FreeNA [47] and effective number of alleles was calculated with GenAlEx 6 [48] using default settings.

## Competing interests

The authors declare that they have no competing interests.

## Authors’ contributions

AH participated in the design of the study, carried out the primer design and amplification of the candidate genes, analyzed bioinformatic as well as genetic data and drafted the manuscript. TE developed the allele finding algorithm. DK worked on SQL scripting and pipeline design. MB participated in primer design and candidate gene amplification. KB participated in the design of the study and commented on the manuscript. SF participated in the design of the study and commented on the manuscript. All authors read and approved the final manuscript.

## Supplementary Material

Additional file 1 Self-contained websites displaying alleles and genotypes Click here for file

Additional file 2 **Table S2.**Table displaying linkage disequilibrium among pairwise combined loci Click here for file
