# Supplementary material for: Allele discovery of ten candidate drought-response genes in Austrian oak using a systematically informatics approach based on 454 amplicon sequencing
Source: BMC Res Notes. 2012 Apr 3;5:175. doi: 10.1186/1756-0500-5-175 (PMC3420255; doi:10.1186/1756-0500-5-175)
Supplement: Additional file 1 — Self-contained websites displaying alleles and genotypes [file 1756-0500-5-175-S1.zip › index.html]

Oak genetic data


**Oak genetic data**

Please click a gene name below to access the corresponding alleles:

*ARF16  
BMY7  
ERD8  
LEA14  
LTP  
PER64  
PIP1E  
RD26*

Use the link below to view the genotypes of all available individuals:

Oak genotypes

Alleles are numbered sequentially. To return to this page use the "back" function of your browser.
